
JOURNAL INFORMATION
==============================
NLM Title Abbreviation: ScientificWorldJournal
Journal ID: ScientificWorldJournal
NLM Journal ID: 101131163
Journal ID: TSWJ

The Scientific World Journal

ISSN: 2356-6140
EISSN: 1537-744X
Publisher: Wiley

ARTICLE INFORMATION
==============================
PMCID: PMC6009731
PMID: 29973852
DOI: 10.1100/tsw.2002.298
Article ID: 971654
Article version: 1
Subjects: Editorial

Selected Peer-Reviewed Short Papers from ESF/CSIC Workshop: Analysis, Toxicity and Biodegradation of Organic Pollutants in Groundwater from Contaminated Land, Landfills and Sediments; 8-10 November 2001, Barcelona, Spain

Ockenden Wendy A. *
TheScientificWorld, UK
*Wendy A. Ockenden: wockenden@thescientificworld.com
Publication date: 2002
Electronic publication date: 2002 Apr 12
Volume: 2
First page: 1004
Last page: 1005
Received 2002 Apr 10; Accepted 2002 Apr 10
Copyright: Copyright © 2002 Wendy A. Ockenden.
Copyright year: 2002
License: This is an open access article distributed under the Creative Commons Attribution License, which permits unrestricted use, distribution, and reproduction in any medium, provided the original work is properly cited.
License URL: https://creativecommons.org/licenses/by/3.0/

Keywords: biodegradation, bioremediation, ecotoxicology, bioavailability, groundwater, organic pollutants, pollutant fate and transport, contaminated land, landfill sites, sediment, persistent pollutants, leaching, bioassay

==============================
**Publisher's Note**
Analysis, Toxicity and Biodegradation of Organic Pollutants in Groundwater from Contaminated Land, Landfills and Sediments
*TheScientificWorldJOURNAL* (2002) 2, 1004–1005
ISSN 1537-744X; DOI 10.1100/tsw.2002.298

### Selected Peer-Reviewed Short Papers from ESF/CSIC Workshop: *Analysis, Toxicity and Biodegradation of Organic Pollutants in Groundwater from Contaminated Land, Landfills and Sediments*; 8–10 November 2001, Barcelona, Spain

Wendy A. Ockenden
*TheScientificWorld U.K.*

*Received April 10, 2002; Accepted April 10, 2002; Published April 12, 2002*

---

**KEY WORDS:** biodegradation, bioremediation, ecotoxicology, bioavailability, groundwater, organic pollutants, pollutant fate and transport, contaminated land, landfill sites, sediment, persistent pollutants, leaching, bioassay

**DOMAINS:** environmental chemistry, bioremediation and bioavailability, soil systems

---

The workshop *Analysis, Toxicity and Biodegradation of Organic Pollutants in Groundwater from Contaminated Land, Landfills and Sediments* was held in Barcelona, Spain 8–10 November 2001. GPoll of European Science Foundation (ESF) and Consejo Superior de Investigaciones Cientificas (CSIC) organized the workshop.

The analysis, fate and effects of organic pollutants in groundwater from diffuse, local, and subsurface infrastructure pollution sources were explored by the workshop. Oral and poster presentations covered various topics, including:

- Identification of major organic pollutants in groundwater resources from contaminated land, landfill, and sediments
- Sample preparation methods for groundwater organic pollutants, including Accelerated Solvent Extraction and Microwave Assisted Extraction
- Advanced analytical methods, including GC-MS and LC-MS
- Use of biosensors as rapid screening systems for the determination of target organic pollutants and their effects
- Evaluation of biosensors compared with current analytical techniques
- Genotoxicity measurements and receptor assays to assess the impact of organic pollutants on the ecosystem and different organisms
- Biodegradation of organic pollutants
- Risk assessment

*Corresponding author. Email: wockenden@thescientificworld.com
©2002 with author.

TheScientificWorld is pleased to announce the forthcoming publication of a collection of selected peer-reviewed short communication papers from the workshop in *TheScientificWorldJOURNAL*. Guest Editors of the collection are Damià Barceló, Jordi Dachs, and Mira Petrović, IIQAB-CSIC, Barcelona, Spain. Damià Barceló was Chair of the Coordinating Committee and Mira Petrović was Scientific Secretary. Jordi Dachs was a Member of the Coordinating Committee. Papers will be published immediately on peer review and acceptance.

The publisher wishes to thank the guest editors, authors and reviewers for their help in the publication of this collection.

Wendy A. Ockenden
10 April 2002

---

**This article should be referenced as follows:**

Ockenden, W.A. (2002) Selected peer-reviewed short papers from ESF/CSIC workshop: Analysis, Toxicity and Biodegradation of Organic Pollutants in Groundwater from Contaminated Land, Landfills and Sediments; 8–10 November 2001, Barcelona, Spain. In Analysis, Toxicity and Biodegradation of Organic Pollutants in Groundwater from Contaminated Land, Landfills and Sediments. *TheScientificWorldJOURNAL* **2**, 1004–1005.

---
